# *De novo* assembly and characterization of fruit transcriptome in *Litchi chinensis* Sonn and analysis of differentially regulated genes in fruit in response to shading

**DOI:** 10.1186/1471-2164-14-552

**Published:** 2013-08-14

**Authors:** Caiqin Li, Yan Wang, Xuming Huang, Jiang Li, Huicong Wang, Jianguo Li

**Affiliations:** 1 China Litchi Research Center, South China Agricultural University, Guangzhou 510642, China; 2 Beijing Genomics Institute at Shenzhen, Shenzhen 518083, China

**Keywords:** *Litchi chinensis*, Transcriptome, Fruit, RNA-Seq, DTA, Shade, Abscission

## Abstract

**Background:**

Litchi (*Litchi chinensis* Sonn.) is one of the most important fruit trees cultivated in tropical and subtropical areas. However, a lack of transcriptomic and genomic information hinders our understanding of the molecular mechanisms underlying fruit set and fruit development in litchi. Shading during early fruit development decreases fruit growth and induces fruit abscission. Here, high-throughput RNA sequencing (RNA-Seq) was employed for the *de novo* assembly and characterization of the fruit transcriptome in litchi, and differentially regulated genes, which are responsive to shading, were also investigated using digital transcript abundance(DTA)profiling.

**Results:**

More than 53 million paired-end reads were generated and assembled into 57,050 unigenes with an average length of 601 bp. These unigenes were annotated by querying against various public databases, with 34,029 unigenes found to be homologous to genes in the NCBI GenBank database and 22,945 unigenes annotated based on known proteins in the Swiss-Prot database. In further orthologous analyses, 5,885 unigenes were assigned with one or more Gene Ontology terms, 10,234 hits were aligned to the 24 Clusters of Orthologous Groups classifications and 15,330 unigenes were classified into 266 Kyoto Encyclopedia of Genes and Genomes pathways. Based on the newly assembled transcriptome, the DTA profiling approach was applied to investigate the differentially expressed genes related to shading stress. A total of 3.6 million and 3.5 million high-quality tags were generated from shaded and non-shaded libraries, respectively. As many as 1,039 unigenes were shown to be significantly differentially regulated. Eleven of the 14 differentially regulated unigenes, which were randomly selected for more detailed expression comparison during the course of shading treatment, were identified as being likely to be involved in the process of fruitlet abscission in litchi.

**Conclusions:**

The assembled transcriptome of litchi fruit provides a global description of expressed genes in litchi fruit development, and could serve as an ideal repository for future functional characterization of specific genes. The DTA analysis revealed that more than 1000 differentially regulated unigenes respond to the shading signal, some of which might be involved in the fruitlet abscission process in litchi, shedding new light on the molecular mechanisms underlying organ abscission.

## Background

Litchi (*Litchi chinensis* Sonn.), one of the most important tropical and subtropical plants in the Sapindaceae family was originated in China, where it has been cultivated for more than 2,300 years [1]. The Sapindaceae is a relatively large family, including at least 125 genera and 1,000 species, which are widely distributed in the tropical and subtropical areas. Being the most widely cultivated fruit crop in this family, litchi becomes a significant contribution to the lives and economies of millions of people in Southeast Asia. In 2010, litchi production in China was 1,780,000 tons from 553,000 ha [2], providing major employment for the local people. The desirable characteristics of litchi fruit, such as a bright colour, exotic aroma, excellent flavour and rich nutrients, make it very attractive and popular in the international markets [3,4]. Despite the economic and commercial importance of litchi, there have been few genomic studies on this crop. This is confirmed by the very few number of litchi gene sequences available in public databases, with only 354 nucleotide sequences available in the NCBI GenBank as of 6 Augest 2013 (http://www.ncbi.nlm.nih.gov/GenBank/). The lack of sequence data for the species of the Sapindaceae family has greatly hindered their research at the molecular level.

Next generation sequencing technology (NGS), such as high-throughput paired-end RNA sequencing (RNA-Seq) and digital transcript abundance(DTA)tag profiling, has greatly facilitated investigation of the functional complexity of transcriptomes for non-model organisms without a reference genome [5-7]. *De novo* assembly of a transcriptome from RNA-Seq produces a genome-scale transcription map that contains both the transcriptional structure and expression level for each gene.

Even though RNA-Seq and DTA have not yet been performed with the members of the Sapindaceae family, they have been widely applied to many other fruit crops for transcriptome analysis, gene discovery and molecular marker development. For examples: more than 59 million reads were generated and 6,695 unigenes were expressed in a stage-specific manner in three different developmental stages of berries in *Vitis vinifera*[8]; 9,839 transcript assemblies were obtained and about 115 simple sequence repeat markers were identified in pomegranate (*Punica granatum*) [9]; 41,239 unigenes with a mean length of 531 bp were *de novo* assembled and more than 3,600 unigenes were differentially expressed in three ripening stages in Chinese bayberry (*Myrica rubra*) [10]. In *Siraitia grosvenorii*, RNA-Seq was combined with DTA to understand mogroside biosynthesis. About 49 million high-quality reads were assembled into 43,891 unigenes, in which 85 cytochrome P450 and 90 UDP-glucosyltransferase unigenes were identified, and seven and five of these unigenes, respectively, were found to be involved in mogroside biosynthesis [11].

It is well known that litchi trees generally produce many more female flowers than necessary [12]. Most of the inevitable massive abscission of flowers and fruitlet occurs after pollination, leading to a low fruit set [13]. To date, rather than investigating at the molecular level, most biological studies on abscission in litchi have been carried out on the physiological level related to nutrients and hormones [14,15].

During early fruit development, active sinks such as the growing shoots and fruit compete for limited carbohydrate and nutrient resources [16]. Artificial shading over the whole canopy is a practical research approach used to induce fruit abscission and investigate its various underlying physiological and molecular mechanisms. Shading during this period not only rapidly decreases photosynthesis, reducing the availability of assimilates but further aggravates the competition among these sinks [17-19]. A decrease in the relative growth rates of fruit is apparent within 2 days after shading [18,19]. Fruit abscission begins at 5 to 10 days after shading and peaks at 15 days [20-22]. Therefore, reduction in fruit growth is an earlier response to shading, while fruit abscission is the final effect of shading on fruit. Severe fruitlet abscission usually leads to a very low yield and causes significant economic losses for farmers.

During the last few years, several methods have been used to study the transcriptional regulation of fruit abscission, including suppression subtractive hybridization and cDNA-amplified fragment length polymorphism, as well as microarrays. With suppression subtractive hybridization technology, a total of 347 expressed sequence tags were obtained and only 112 unigenes were found in the cDNA library of fruitlet, in apples (*Malus × domestica*), after shading [23]. cDNA-amplified fragment length polymorphism identified 227 differentially expressed clones isolated from the seeds, cortices and peduncles of abscising fruitlet and non-abscising fruitlet populations[24]. Because of the limited throughput and high false-positive rate of the two methods mentioned above, microarray was applied to several species to obtain large-scale transcriptional regulation information during organ abscission in apples [22,25], citrus (*Citrus clementina*) [26] and tomato (*Solanum lycopersicum*) [27]. Recently, Gil-Amado and Gomez-Jimenez [28] compared the olive fruit abscission zone transcriptomes at two different stages (pre-abscission versus abscission) using the RNA-Seq technique; 148 Mb of sequences (443,811 good-quality sequence reads) were obtained and 4,728 differentially expressed genes were identified from these two samples. Of these 4,728 differentially expressed genes, 2,314 were up-regulated and 2,414 were down-regulated at the abscission stage in the fruit abscission zone [28].

RNA-Seq is a fast and comprehensive approach for direct sequencing at an extraordinary depth, while DTA is based on sequencing serial analysis of gene- expression libraries and generates a digital output proportional to the number of transcripts per mRNA [29]. Over the last few years, studies in marine fish (*Lateolabrax japonicus*) [30], whitefly (*Bemisia tabaci*) [31] and *Siraitia grosvenorii*[11] have demonstrated that the combination of these two technologies is very suitable for studying transcriptome profiles and provides a good understanding of the complexity of gene expression, regulation and networks. However, none of the techniques mentioned above have been applied in litchi to date, simply because of the lack of genomic sequence information. In this study, the litchi fruit transcriptome was first *de novo* assembled and annotated using RNA-Seq and various computation tools, following which large-scale differentially expressed transcripts in response to shading were explored using DTA. This information will be very helpful for improving the functional genomics studies in litchi and furthering our understanding of the molecular mechanisms behind shade-induced fruit abscission in fruit trees.

## Results

### Paired-end sequencing and *de novo* assembly

A pooled cDNA sample representing diverse tissues and developmental stages of litchi fruit was prepared and sequenced with Illumina paired-end sequencing. After a stringent quality check and data cleaning, 53,437,444 paired-end reads with a total of 4,007,808,300 bp were obtained and assembled into 57,050 unigenes (Table 1). The quality of the reads was assessed using the base-calling quality scores from the Illumina’s base-caller Bustard. In addition, 95.79% of the clean reads had Phred-like quality scores at the Q20 level (a sequencing error probability of 0.01). Using the SOAPdenovo software (http://soap.genomics.org.cn), a total of 134,593 contigs (>100 bp) with an average length of 278 bp, 96,711 scaffolds (>100 bp) with an average length of 410 bp, and 57,050 unigenes(>200 bp) with a mean length of 601 bp were assembled, respectively (Table 1). The length distributions of the contigs, scaffolds and unigenes are listed in Additional file 1. Taken unigenes as an example, the lengths of unigenes ranged from 200 to 10,687 bp. Of the 57,050 unigenes, 37,290 unigenes (65.36%) were between 200 and 500 bp; 11,687 unigenes (20.49%) ranged from 501 to 1,000 bp; and 8,073 unigenes (14.15%) were larger than 1,000 bp (Additional file 1). In addition, 76,567 scaffolds (79.17%) and 44,158 unigenes (77.40%) showed no gaps (Additional file 2).

**Table 1:** Summary of the litchi fruit transcriptome

**Category**	**Number**	**N50 (bp)**	**Mean length (bp)**	**Total nucleotides (bp)**
Reads	53,437,444	75	75	4,007,808,300
Contig(≥100bp)	134,593	365	278	37,365,463
Scaffolds(≥100bp)	96,771	643	410	39,705,034
Unigenes(≥200bp)	57,050	811	601	34,312,642

N50 = median length of all non-redundant sequences.

### Functional annotation and classification

**Figure 1:**
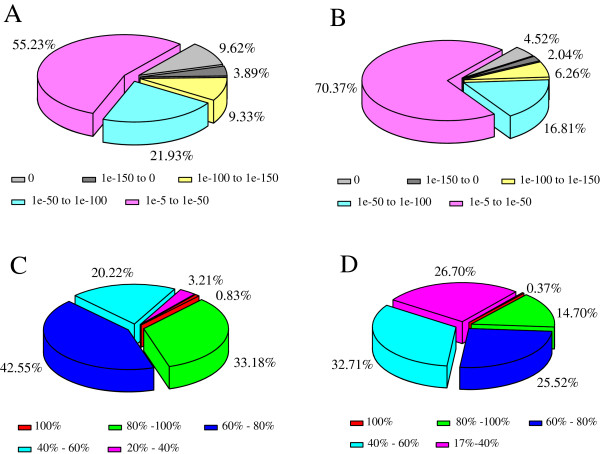
**Characteristics of homology search of assembled unigenes against the NCBI NR and Swiss-Prot databases. ***E*-value distribution of the top BLAST hits for each unigene with a cut-off *E*-value of 1.0E-5 in **(A)** the NCBI non-redundant (NR) database and **(B)** the Swiss-Prot database. Similarity distribution of BLAST hits for each unigene in **(C)** the NR database and **(D)** the Swiss-Prot database.

For annotation, the assembled unigenes were first compared against the NCBI non-redundant (NR) and the Swiss-Prot protein databases using BLASTx analysis with a cut-off *E*-value of 10^–5^. The results indicated that 34,029 of the 57,050 unigenes (59.65%) could be annotated based on sequences in the NR database, while 22,945 unigenes (40.22%) were aligned to known proteins in the Swiss-Prot database (Table 2). To further analyze the BLAST results, the *E*-value and similarity distributions were calculated (Figure 1). Statistical analysis of the top hits in the NR database showed that 44.77% of the mapped sequences had significant homology (<1.0E-50), and almost 34.01% of the sequences had alignment identities greater than 80% (Figure 1A and 1C). As expected, a comparable pattern of *E*-value and similarity distribution of the top BLAST hits was found in the Swiss-Prot database. Figure 1B and 1D show that 29.63% and 15.07% of the mapped sequences, respectively, had significant homologies and similarities higher than 80% in the Swiss-Prot database.

**Table 2:** Summary of functional annotation of assembled unigenes

**Public protein database**	**Number of unigene hits**	Percentage (%)*
NCBI NR	34,029	59.65%
Swiss-Prot	22,945	40.22%
GO	5,885	10.32%
COG	10,234	17.94%
KEGG	15,330	26.87%

* Proportion of the 57,050 assembled unigenes.

**Figure 2:**
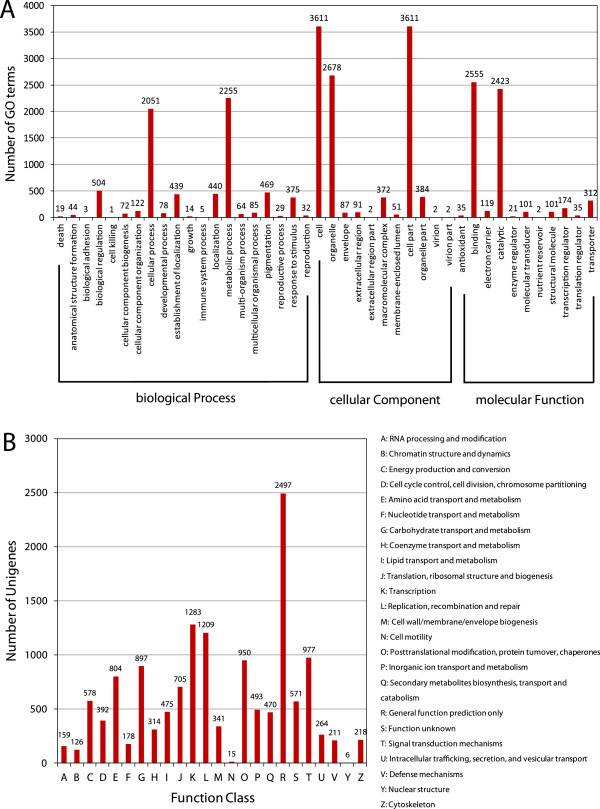
**Functional classification. (A)** Functional classification of assembled unigenes based on Gene Ontology (GO) categorization. The results are summarized in three main GO categories: biological process, cellular component and molecular function. The x-axis indicates the subcategories and the y-axis indicates the numbers related to the total GO terms present; the unigene numbers assigned the same GO terms are indicated on the top of the bars. **(B)** Histogram of Clusters of Orthologous Groups (COG) classification. All unigenes were aligned to the COG database to predict and classify possible functions. Out of 34,029 hits in the NCBI non-redundant (NR) database, 10,234 unigenes were annotated and separated into 24 clusters.

On the basis of NR annotation, Gene Ontology (GO) analysis was performed. Of the 34,029 annotated unigenes, 5,885 sequences were assigned with one or more GO terms (Table 2). These 5,885 unigenes were categorized into 42 GO functional groups (http://www.geneontology.org), which are distributed under the three main categories: molecular function (5,878), biological process (7,101) and cellular components (10,891) (Figure 2A). Within the molecular function category, genes encoding binding proteins (43.47%) and proteins related to catalytic activity (41.22%) were the most enriched. Proteins related to metabolic processes (31.76%) and cellular processes (28.88%) were enriched in the biological process category. Under the cellular components category, the cell (33.16%), cell part (33.16%) and organelle (24.59%) were the most highly represented GO terms.

In addition to GO analysis, Clusters of Orthologous Groups (COG) analysis was used to further evaluate the function of our assembled unigenes. In total, out of 34,029 NR hits, 10,234 hits were aligned with the 24 COG classifications (Table 2). In the 24 COG categories, the cluster for ‘general function prediction only’ (24.40%) represented the largest group, followed by ‘transcription’ (12.54%) and ‘replication, recombination and repair’ (11.81%). Only a small portion of the unigenes were assigned to ‘nuclear structure’ (0.059%) or ‘cell motility’ (0.15%). It is worth noting that a large number of genes were assigned to ‘signal transduction mechanisms’ (9.55%), ‘carbohydrate transport and metabolism’ (8.76%) and ‘cell wall/membrane/envelope biogenesis’ (3.33%) (Figure 2B).

**Figure 3:**
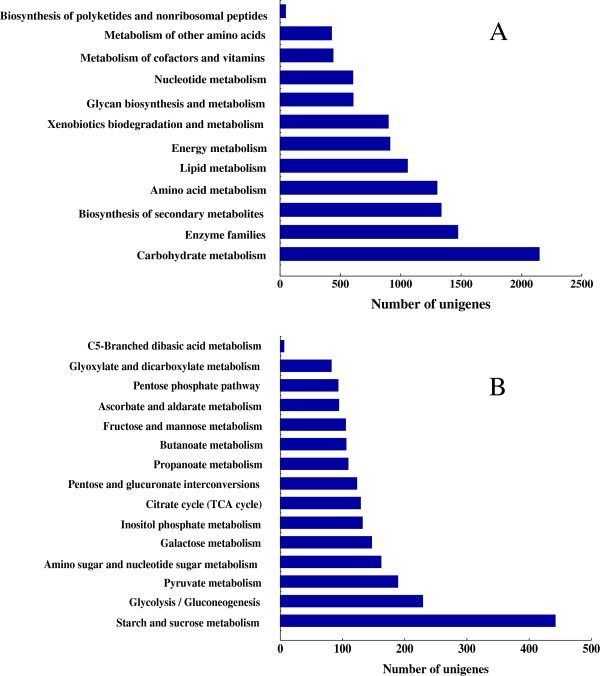
**Pathway assignment based on the Kyoto Encyclopedia of Genes and Genomes (KEGG). (A)** Classification based on metabolism categories; **(B)** Classification based on carbohydrate metabolism categories.

To explore the pathways in which these annotated genes were involved, the Kyoto Encyclopedia of Genes and Genomes (KEGG) analysis with an *E*-value cut-off less than 10^-5^ was also conducted. A total of 15,330 (26.87%) annotated unigenes had significant matches with 26,311 hits in the KEGG database and were assigned to 266 KEGG pathways (Table 2). Among them, about 11,244 unigenes were assigned to metabolic pathways. As shown in Figure 3A, 2,148 unigenes were clustered into carbohydrate metabolism, followed by enzyme families (1,473 unigenes), biosynthesis of secondary metabolites (1,335 unigenes) and amino acid metabolism (1,301 unigenes), and so on. Carbohydrate metabolism can be further classified into 15 subcategories (Figure 3B); most of which were mapped to pathways of starch and sucrose metabolism, glycolysis/gluconeogenesis, pyruvate metabolism, amino sugar and nucleotide sugar metabolism, or galactose metabolism.

### Evaluation of the DTA library and sequencing

To explore the differentially expressed genes induced by shading treatment, DTA profiling was conducted for both shaded and non-shaded samples. After data cleaning, a total of 3.6 million and 3.5 million high-quality tags, representing 126,156 and 128,505 tag entities with unique nucleotide sequences (distinct tags) were generated from shaded and non-shaded libraries respectively. Among these, 58,758 and 59,691 distinct tags identified in 24,181 and 23,881 unigenes, respectively, were unambiguously mapped to the litchi reference transcriptome database obtained in this study (Additional file 3). The distribution of total tags and distinct tags throughout different tag abundance categories showed significantly similar patterns in these two libraries (Additional file 4).

### Differentially expressed unigenes between shaded and non-shaded libraries

**Figure 4:**
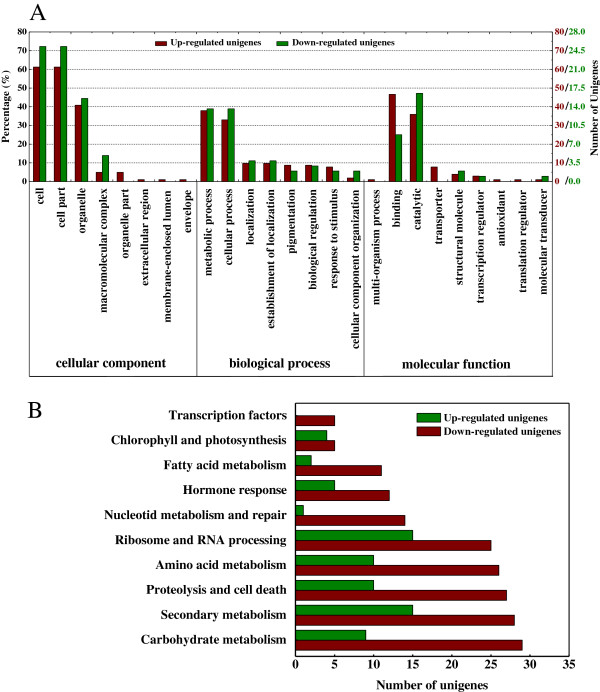
**Proportions of and comparisons between up- and down-regulated unigenes. (A)** Function classifications based on Gene Ontology (GO). The results are summarized in three main categories: cellular component, biological process and molecular function. The left y-axis indicates the percentage of a specific category of unigenes in that main category. The right y-axis indicates the number of unigenes in a category: red (up-regulated unigenes) or green (down-regulated unigenes). **(B)** Pathway assignment based on the Kyoto Encyclopedia of Genes and Genomes (KEGG) with classification based on metabolism categories. The bottom x-axis indicates the percentage of a specific category of unigenes in that main category. The top x-axis indicates the number of unigenes in a category: red (up-regulated unigenes) or green (down-regulated unigenes).

To reveal the molecular events behind the DTA profiles, we analyzed the differentially expressed unigenes between shaded and non-shaded libraries. In our study, more than 99% of the distinct tag entities showed the expression within a five-fold change between the two libraries (Additional file 5). To identify candidate unigenes that are regulated in response to shading, an algorithm developed by Audic and Claverie [32] was used to compare their transcript abundance profiles. A total of 3,209 regulated tag entities, which were mapped to 1,039 unigenes, showed significant differential expression (false discovery rate [FDR]≤ 0.001, |log_2_ ratio| ≥1, Additional file 6A); among these, 757 and 282 unigenes were up- and down-regulated, respectively (Additional file 6B). However, only 175 unigenes were functionally annotated with GO terms (Figure 4A) and 405 unigenes were identified in the KEGG pathway annotation (Figure 4B). Interestingly, approximately 33.8% of the unigenes with known function fell into the categories related to carbohydrate metabolism, proteolysis and cell death, hormone response, amino acid metabolism or photosynthesis (Figure 4B), suggesting that these pathways or processes might respond to the shading signal.

**Figure 5:**
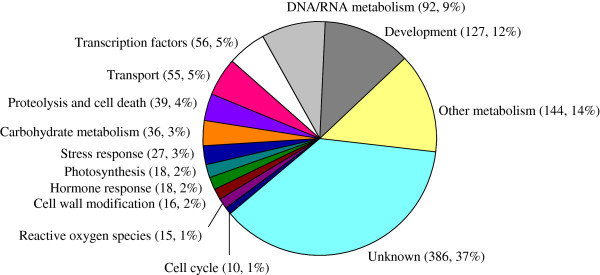
**Distribution of statistically significant unigenes for shading-treated fruitlet.** 1039 significant unigenes were classified into 14 functional categories. Two numbers in parentheses represent the number of unigenes in each category and the percentage of them to the total, respectively.

Since GO categories and KEGG annotation are too general to provide detailed information on the biological mechanisms, additional annotation (NR databases) was further conducted on all of 1,039 unigenes with differential expression patterns. These unigenes were divided into 14 groups (Figure 5, Additional file 7). Ten groups (photosynthesis, carbohydrate metabolism, transport, transcription factors, hormone response, stress response, cell wall modification, hydrolysis and cell death, reactive oxygen species [ROS] and cell cycle) accounted for only about 28% of the differential unigenes; one unknown group consisted of about 37% of the significant unigenes, and the remaining three groups (development, DNA/RNA and the other metabolism, such as secondary metabolism, nitrogen metabolism, amino acid metabolism and so on) took up approximately the other 35% of the unigenes.

### Effects of shading on fruitlet abscission and expression of selected differentially regulated genes between two TDA libraries

**Figure 6:**
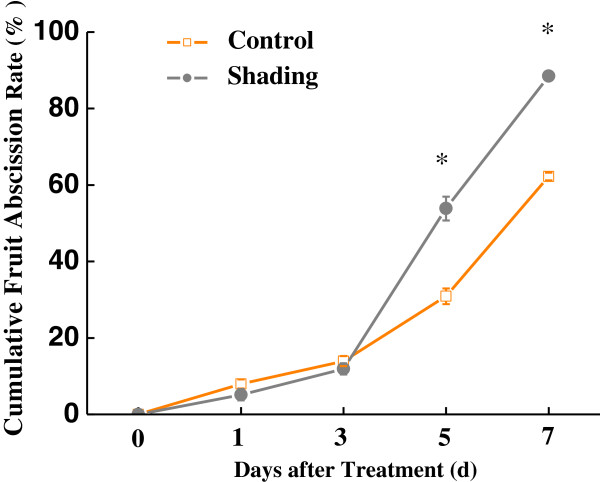
**Fruitlet abscission in shaded and non-shaded trees of litchi cv. ‘Nuomici’ (2009).** The orange line is non-shading control, and the grey line is shading treatment (shaded to 18% of full sun with a neutral density black-polypropylene shade cloth). Shading treatment lasted 7 days since 30 days after anthesis. Bars indicate SE for the mean value of the cumulative fruitlet abscission percentage of three biological replicates. Significances at 5% level are indicated with stars (‘*’) according to t-test.

To uncover the roles of the differentially expressed unigenes mentioned above, the effects of shading on fruitlet abscission and the expression of randomly selected differentially regulated genes between two DTA libraries were further studied. The cumulative fruit abscission rates (CFARs) were calculated and compared among the shaded and non-shaded trees. The CFARs showed similar trends (Figure 6), which gradually increased during the first 3 days. Four days after treatment, the CFAR in shade-treated fruits was significantly higher than that in the non-shaded controls. Consequently, 88.5% of the fruit on the shaded trees dropped 7 days after treatment, compared with a 62.2% loss among controls, indicating that shading treatment significantly intensified fruitlet drop.

**Figure 7:**
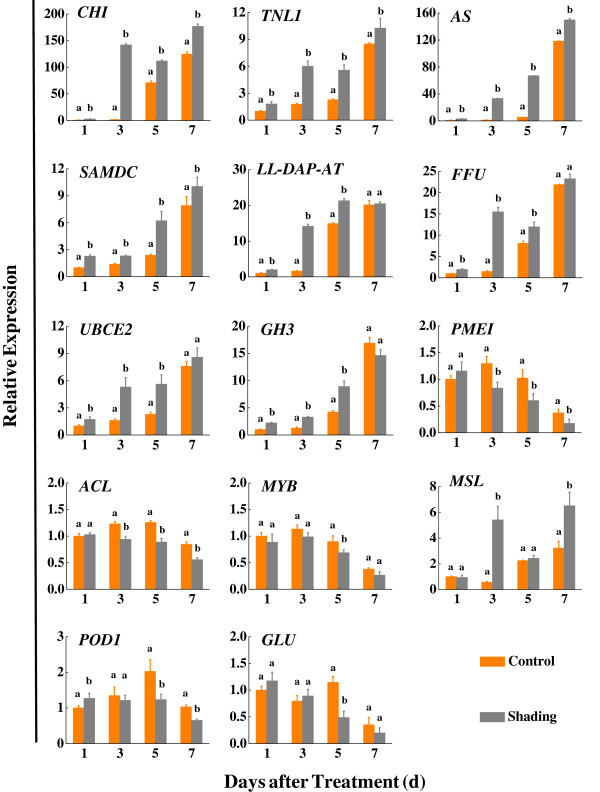
**qRT-PCR analysis of 14 randomly selected genes in fruits from control (orange bars) and shaded (grey bars) trees.** Genes up-regulated and down-regulated by shading treatment. *EF-1α* was used as a reference gene for normalization of gene-expression data. Data show expression of a gene relative to its expression at 1 day after treatment in control fruits. Error bars indicate standard errors of the means (n=3). Bars with different lower-case letters indicate significant differences based on a t-test at the P ≤0.05 level. *CHI*: Chitinase; *TNL1*: Thioredoxin-like 1; *AS*: Asparagine synthase; *SAMDC*: S-adenosylmethionine decarboxylase; *LL-DAP-AT*: LL-diaminopimelate aminotransferase; *FFU*: *β*-fructofuranosidase; *UBCE2*: Ubiquitin-conjugating enzyme E2; *GH3*: Indole-3-acetic acid-amido synthetase; *PMEI*: Invertase/pectin methylesterase inhibitor; *ACL*: ATP citrate lyase; *MYB*: MYB family transcription factor; *MSL*: Mannose/ glucose-specific lectin; *POD1*: Peroxidase 1; *GLU*: Endo-*β*-1, 3- glucosidase.

Fourteen genes were randomly selected in order to compare their expression patterns by quantitative real-time PCR (qRT-PCR) with fruitlet abscission patterns during shading treatment (Additional file 8). Four genes, encoding *β*-fructofuranosidase (*FFU*), endo-*β*-1,3-glucosidase (*GLU*), invertase/pectin methylesterase inhibitor (*PMEI*) and ATP citrate lyase (*ACL*), were related to carbohydrate metabolism, while another four genes, encoding chitinase (*CHI*), mannose/ glucose-specific lectin (*MSL*), thioredoxin-like 1 (*TNL1*) and peroxidase 1 (*POD1*), were involved in pathogen defence and oxidation-reduction reaction. The remaining five genes, encoding ubiquitin-conjugating enzyme E2 (*UBCE2*), asparagine synthase (*AS*), indole-3-acetic acid-amido synthetase (*GH3*), LL-diaminopimelate aminotransferase (*LL-DAP-AT*) and *S*-adenosylmethionine decarboxylase (*SAMDC*), were involved in ubiquitination, nitrogen metabolism, hormone metabolism, amino acid metabolism and polyamine synthesis respectively. And lastly, the MYB family transcription factor (*MYB*) belongs to the transcription factors directly involved in gene expression control. As listed in Additional file 8, nine and five unigenes were up- and down-regulated respectively. The qRT-PCR results showed a similar change trend for all tested unigenes in the shaded and non-shaded fruits (Figure 7). The mRNA transcript abundance of eight unigenes (*CHI*, *TNL1*, *AS*, *SAMDC*, *LL-DAP-AT*, *FFU*, *UBCE2* and *GH3*) constantly increased after treatment, with significantly higher expression compared to the non-shaded control, except for four unigenes (*LL-DAP-AT*, *FFU*, *UBCE2* and *GH3*) at 7 days after treatment. Three unigenes (*PMEI*, *ACL* and *MYB*) exhibited consistently significant down-regulated expression under shaded treatment, which represented a trend opposite to the fruitlet drop patterns from day 3 to day 7 after shading. The previously mentioned eleven unigenes might be associated with fruitlet abscission induced by shading. The remaining three unigenes (*MSL*, *POD1* and *GLU*) might not be directly related to fruit shedding, as indicated by their irregular expression patterns between shaded and non-shaded trees throughout the whole experiment.

## Discussion

### First litchi fruit reference transcriptome generated by RNA-Seq

Computer-based *de novo* assembly tools (e.g., Trans-ABySS, Velvet-Oases, SOAPdenovo, Trinity) have been developed in conjunction with massively parallel sequencing and their usefulness in transcriptome assembly has been demonstrated in previous studies. Garg *et al.* found that transcriptome assembly with Velvet followed by Oases program was better than that with other programs based on various assessment parameters (e.g., N50 length, average contig length and sequence similarity with closely related species) [33]. It was also noted that Oases was the most appropriate program for *de novo* assembly of the wheat (*triticum aestivum*) transcriptome, when compared with Trans-ABySS and Trinity [34].

Only the SOAPdenovo program was used for sequence assembly in the current study since many studies have demonstrated that SOAPdenovo is a powerful tool for annotating transcriptomes for plant species across taxa, including sweet potato (*Ipomoea batatas*) [35], Chinese bayberry (*Myrica rubra*) [10], *Siraitia grosvenorii*[11], *Taxus mairei*[36], *Hevea brasiliensis*[37], *Salvia miltiorrhiza*[38], citrus [39], *Momordica cochinchinensis*[40] and peanut (*Arachis hypogaea*) [41]. In this study, a total of 4,007,808,300 bp were sequenced and assembled into a reference transcriptome of litchi fruit, which is also the first library in the Sapindaceae family to be reported. Using the SOAP*denovo* software, we generated a total of 57,050 unigenes (≥200 bp), whose average length (601 bp) was longer than that documented in other studies using similar technology and different research organisms, such as *Momordica cochinchinensis* (388 bp) [40], *Hevea brasiliensis* (436 bp) [37], sweet potato (581 bp) [35], *Salvia miltiorrhiza* (467 bp) [38] and Chinese bayberry (531 bp) [10]. Our results showed that more than 59% of unigenes were matched with functional annotations in the NR database, which is comparable with results from other studies using the same approach (e.g., >61% in *Salvia miltiorrhiza*[38], 43% in *Momordica cochinchinensis*[40] and 58% in *Hevea brasiliensis*[37]). We found that more unigenes are involved in the starch and sucrose metabolism pathways than in other carbohydrate metabolism pathways that are active in the fruit. These results are consistent with the findings in *Siraitia grosvenorii*[11], Chinese bayberry [10] and date palm (*Phoenix dactylifera*) [42]. Taken together, the litchi fruit reference transcriptome assembled in this study is comprehensive, accurate and useful for future genetic research of litchi fruit.

### Discovery of more than 1,000 unigenes differentially regulated in fruit in response to shading using the DTA method

Fruit is an organ that depends mainly on nutrition imported from elsewhere in the plant, especially photosynthates from leaves [43]. Shading during early fruit development decreases fruit growth and induces fruit abscission by reducing the availability of assimilates [17]. Although many researchers have made great efforts to elucidate the mechanisms of shading effects on fruit growth and abscission, the symptomatic molecular events related to this treatment are still largely unknown [19,22,23,44]. By employing the method of suppression subtractive hybridization, Zhou *et al*. isolated, from apple fruitlet, 112 shade-response unigenes belonging to eight functional categories [23]. Through the use of apple oligonucleotide microarray, 1,057 selected genes belonging to 15 functional categories, from shade-treated fruit abscission zone, were discovered [22]. In this study, we discovered that the expression of 1,039 unigenes, which fell into 14 groups, was impacted by shading.

A reduction in light intensity is thought to be the most direct effect of shading. It has been hypothesized that photosynthetic rates are significantly altered by different levels of shading [45,46]. Zhu *et al*. found that about 6% of shade-response genes are related to photosynthesis and more than 90% of these genes are repressed in the apple fruit abscission zone [22]. In the current study, about 2% of litchi unigenes induced by shading were found to be related to photosynthesis, and only 28% of the genes documented were repressed. The repressed unigenes were mainly involved in light harvesting and electron transport for photosystem I, while the remaining up-regulated unigenes included genes involved in chlorophyll biosynthesis, light harvesting and electron transport for photosystem II, and the chlorophyll DNA binding genes. Fruit setting and development are highly dependent on the carbohydrate supply. In response to shading, 36 differentially expressed unigenes encode enzymes involved in carbohydrate metabolisms, such as glycosidases, hydrolases, and transferases, most were up-regulated, which is in agreement with the findings in apples [22,23]. The up-regulated expression of these genes is likely a direct or indirect early response of the fruitlet to the carbohydrate shortage.

Shading during early fruit development retards fruit growth by decreasing cell division and expansion [44]. Reductions in cell division have been associated with coordinated alterations in the expression of some key cell-cycle genes [44,47]. Even though the current study found that only CDK inhibitor was up-regulated, it also showed that six cell-cycle-related unigenes were repressed by shading, including cyclin-dependent kinase (CDK), cyclin-dependent kinase-activating kinase (CAK) and some cell-division control proteins. These results are also consistent with those from previous studies in apples [22,44].

Some research groups have exploited the fact that dark-induced cell death is usually accompanied by chlorophyll degradation, protein hydrolysis, ROS generation, and cell-wall modification [48,49]. In this study, eight unigenes related to cell death were up-regulated by shading, particularly cysteine protease and senescence-associated and autophagy-related genes. Shading also had a large impact on protein ubiquitination and degradation; for example, F-box proteins, 26S proteasome subunit proteins, ubiquitin E3 ligase complex including cullin and ubiquitin-conjugating enzymes were positively regulated in response to shading, which is in agreement with the findings in apples [22]. Different levels of dark-induced ROS production have also been reported during the senescence process [49-51]. Only a core NADPH oxidase unigene was increased in the shaded litchi fruitlet. In contrast, some unigenes, involving in the cellular response to oxidative stress, were induced such as a glutathione S-transferase, two glutaredoxin and six thioredoxin genes. It has been previously pointed out that shading has an effect on cell-wall loosening and degradation [22,23]. However, in our study, only two cell-wall degradation related genes (polygalacturonase and β-d-galactosidase) were induced after shading treatment. Three cellulose synthases, two cell-wall structure genes and a single callose synthase, involved in the secondary cell-wall formation and stress resistance [52-54] were induced. These results indicated that fruits active cellular oxidation resistance and cell-wall formation in response to low-light stress. More than 92% of the differentially expressed unigenes related to stress response were up-regulated, including a large group of resistant genes against diverse stress conditions in this study.

A complex combination of signalling pathways has been proposed to play important roles in the modulation of fruit development. Hormone signalling is key for the coordination of fruit growth, fruit abscission and plant defence [27,55,56]. As many as 18 differentially expressed unigenes were hormone-related and involved in the signal transduction pathways of auxins, brassinosteroids, gibberellins, jasmonic acid and polyamine. All of them were up-regulated, except for a single auxin efflux carrier gene, in response to the shading treatment. It is puzzling that no unigene, related to abscisic acid (ABA), ethylene biosynthesis and signalling, was found to be significantly changed by shading. In fact, ABA and ethylene have been implicated in the regulation of stress-induced senescence. Several genes involved in ABA and ethylene biosynthesis and signalling were induced in the shading-treated fruit abscission zone in apples [22]. However, several groups of transcription factors associated with senescence and stress, such as *AP2/ERF*, *NAC*, *WRKY* and zinc finger (C_3_HC_4_ and C_2_H_2_ type) families, were all largely increased in the shaded fruits, which is consistent with the results on leaf senescence in *Arabidopsis* by shading [57]. Another group of differentially expressed genes involved in ATP-binding cassette, calcium binding and membrane transport, as well as those involved in the transportation of sugars, amino acids, nucleosides, proteins, lipids, anions and cations. As previously mentioned above, shading during early fruit development reduces canopy photosynthesis and enhances competition between fruits. This results in the alteration of available carbohydrate and transportation of nutrients in favour of shoot growth and at the expense of fruit development [19,58]. Therefore, 41 out of 55 transcription factors were positively induced by shading in our study, which might reflect the shifting function of the shaded fruitlet, as they become a source tissue for the mobilization of nutrients to other vegetative organs.

### Differentially regulated genes involved in litchi fruitlet abscission in response to shading

The imposition of shading treatment during early fruit development promotes fruit abscission in several crops including apples [18,20], peaches [59] and grapes [60]. Shade is presumed to induce a transient reduction in the supply of carbohydrates to developing fruit during a period when the fruit is sensitive to such a stress. Shade-induced fruit abscission begins about 5 to 10 days after shading and peaks at 15 days [20-23]. Zhou *et al.*[23] and Zhu *et al.*[22] have exploited dark-induced fruit abscission to characterize many altered metabolic pathways and transcript abundance during this senescence process. In our study, shading treatment significantly stimulated fruit drop from 3 days after treatment and induced 88.5% of the fruit to abscise within 7 days, compared with a 62.2% fruit loss within the same time period in the control. Fourteen differentially regulated unigenes, between shaded and non-shaded DTA-tag profiling libraries, were randomly selected in order to compare their expression patterns with the fruit abscission dynamics during shading treatment. The qRT-PCR results of all the selected up-regulated unigenes obtained on day 3 agreed with the expression levels described by DTA. It is possible that transcriptional changes in response to shading occurred and exerted their effects at the early stage of the abscission process and thus became less apparent after 7 days.

Two factors for comparison between expression profiles were considered: whether the mRNA transcript abundance continued to be generally increased or decreased after days of treatment; and whether the relative expression ratio in samples undergoing shaded treatment was significantly higher than that in the controls at specific sampling dates, particularly at the early stage (1, 3 and 5 days) after treatment. According to these two criteria, 11 of the selected differentially regulated shading-responsive genes are regarded as being involved in the process of fruitlet abscission. Six of these genes are also documented as being related to the shedding of plant organs,including *CHI*[26,27,61], *FFU*[62], *GH3*[63], *SAMDC*[64], *MYB*[28] and *PMEI*[65]. The remaining five genes (*AS*, *ACL*, *TNL1*, *UBCE2* and *LL-DAP-AT*) have not yet been reported in the literature to be involved in organ shedding. The expression of *AS* can be induced in a range of low-carbohydrate situations, such as dark periods and sugar-starved treatment [66,67]. Moreover, an *AS* gene promoter has been identified as a potential carbohydrate-responsive element in *Asparagus*[68]. After shading treatment, the expression of *AS* in litchi fruit was immediately up-regulated and was consistently increased during fruit abscission, indicating that *AS* expression might be a direct effect of the low-carbohydrate conditions. *ACL* catalyzes the ATP-dependant reaction of citrate and coenzyme A to form acetyl- coenzyme A and oxaloacetic acid [69]. In our study, the expression of *ACL* displayed a down-regulated profile after shading treatment, showing that fruit need more energy to maintain the normal physiological functions under low-carbohydrate conditions. Some indirect evidence supported the possibility that ROS generation is related to the organ abscission [26]. Thioredoxin is a ubiquitous small redox active protein in plants that plays a critical role in redox balance regulation through thiol-disulfide exchange reactions. The transcript level of *TNL1* in this study was found to be significantly increased as shading-induced fruit abscission progressed, suggesting that it might be involved in regulating fruit drop by influencing stress responses. The transcript levels of ubiquitin-conjugating enzyme genes (*GhUBC1* and *GhUBC2*) from allotetraploid cotton have been found to increase significantly in leaves and flowers at senescence, indicating that *GhUBC1/2* may play a role in the degradation of target proteins that function in delaying the senescence program [70]. The litchi *UBCE2* unigene in our study was found to be significantly and consistently up-regulated in the shaded fruits, implying that shading-induced fruit abscission induced by shading treatment might be, to some extent, related to the organ senescence and triggered by *UBCE2* up-regulated expression. LL-diaminopimelate aminotransferase, discovered in 2006, is a crucial enzyme in the plant lysine biosynthetic pathway, which converts tetrahydrodipicolinate to LL-diaminopimelate in a single step [71]. Lysine is an amino acid essential for peptidoglycan synthesis. There is information in the literature concerning the relationship between *LL-DAP-AT* or peptidoglycan and organ shedding. However, our study showed that the *LL-DAP-AT* unigene transcript level in shaded fruit continued to be increased and significantly higher in shaded fruit compared with controls from day 1 to day 5 after shading treatment, indicating that *LL-DAP-AT* might have some role in regulating fruit abscission.

It is not surprising that some of the genes, differentially regulated in response to shading, are involved in fruitlet abscission since shading during early fruit development induces an increase in fruitlet drop. However, our study was carried out when fruitlet abscission was occurring, and the expression-pattern analysis of genes in both DTA and qRT-PCR experiments were compared between abscission and increased abscission. This would go some way to making accurate transcriptional changes difficult to measure. Thus, comparing between RNA samples from abscission induced by shading versus no abscission in the control should be suggested in the future experiments to find out the candidate genes specifically responsible for fruitlet abscission in litchi.

## Conclusions

In this report, we present the sequencing, *de novo* assembly and analysis of the fruit transcriptome and provide a global description of transcript abundance profiles in fruits from shaded and unshaded trees using next-generation sequencing technology in *Litchi chinensis*. The transcriptome is described in details in the results section, with an emphasis on annotation using the NR, Swiss-Prot, KEGG and COG databases. We generated more than 53 million paired-end reads and assembled 57,050 unigenes. Using the DTA method, a total of 3.6 million and 3.5 million high-quality tags were obtained from the shaded and non-shaded libraries, respectively. Of these high quality tags, 3,209 regulated tag entities, which were mapped to 1,039 unigenes, showed significant differential expression, suggesting that these potential regulators might function in response to the shading signal. Furthermore, some of shading-induced differentially expressed unigenes were implicated in the fruitlet abscission process in litchi, shedding new light on the molecular mechanisms involved in organ abscission.

## Methods

### Plant materials, sample collection and determination of fruit abscission

Plant materials were collected in 2009 from an orchard located at the South China Agricultural University, Guangzhou, China. For transcriptome sequencing, 16 samples comprising fruitlet (six samples), pericarp (five samples), aril (three samples) and seed (two samples) were collected at different development stages (0, 14, 28, 42, 56, 70 and 84 days after female flower anthesis) from three different litchi cultivars including ‘Hehuadahongli’, ‘Nuomici’ and ‘Heiye’. For DTA library construction, six ‘Nuomici’ litchi trees with uniform vigour and moderate initial crop load were selected. Three were randomly selected as untreated controls while the other three were shaded to 18% of full sun with a neutral-density black-polypropylene shade cloth at 30 days after anthesis. This level of shading has been used to cause fruitlet abscission in many other studies, such as those of apples [23,58]. Each tree was regarded as a biological replicate. Fruitlet samples were collected at 1, 3, 5 and 7 days after treatment from both control and treated trees. To determine the fruit abscission rate, 10 fruit-bearing shoots from each tree were tagged based on the uniformity of the shoots, and the fruit on tagged shoots were counted at 0, 1, 3, 5 and 7 days after treatment. All of the samples mentioned above were immediately frozen in liquid nitrogen and stored at −80°C until use.

### RNA isolation and library construction for transcriptome analysis

Total RNA was isolated using a previously described method [72] and RNA integrity was confirmed using the 2100 Bioanalyzer (Agilent Technologies, Santa Clara, CA, USA). Total RNA of each sample was then pooled with equivalent quantity. After RNA extraction, poly(A)-containing mRNA was purified using oligo(dT) magnetic beads. To avoid priming bias when synthesizing cDNA, the mRNA was first fragmented into small pieces using divalent cations under elevated temperature. The cleaved RNA fragments were then used to synthesize the first-strand cDNA using reverse transcriptase and random primers, followed by second-strand cDNA synthesis using DNA polymerase I and RNase H. Next, these cDNA fragments underwent processes including end repair, addition of a single ‘A’ base and ligation with adapters. Finally, these products were purified and amplified through PCR to create the library for sequencing.

### Transcriptome sequencing, *de novo* assembly and functional annotation

The Solexa HiSeq™ 2000 was employed to sequence the library (BGI, Shenzhen, China). The cDNA fragments were about 200 bp in length and the fragments were sequenced with a paired-end strategy. The raw reads obtained were pre-processed by removing adaptor sequences, and discarding empty reads and low-quality sequences. All the reads were then used for transcriptome *de novo* assembly using the short read assembling program SOAPdenovo (version 1.04; http://soap.genomics.org.cn/soapdenovo.html) [73] at the parameters of “-K 29 -M 2 -L 50” by BGI. Short reads were first assembled into contigs with no gap, and the reads were mapped back to contigs. The resulting contigs were joined into scaffolds using the read mate pairs, with unknown sequences replaced with ‘N’s. Finally, paired-end reads were performed to fill the gap between different scaffolds in order to obtain unigenes with the least ‘N’s and longest sequences. The assembled unigenes were annotated by BLASTx alignment (*E*-value <0.00001) to protein databases such as the NCBI NR protein database (http://www.ncbi.nlm.nih.gov), the Swiss-Prot protein database (http://www.expasy.ch/sprot), the KEGG pathway database (http://www.genome.jp/kegg) and the COG database (http://www.ncbi.nlm.nih.gov/COG). The best-aligning results from the four databases were chosen to decide the sequence direction of unigenes. Concerning the contradictory results between different databases, a priority order of NR, Swiss-Prot, KEGG and COG was considered. As for other sequences that failed to be annotated to any one of the above databases, the ESTScan program (http://www.ch.embnet.org/software/ESTScan.html) was used to predict the ‘CDS’ (coding regions) and their orientations. Functional annotations of unigenes by GO were carried out using the Blast2GO software [74], followed by GO functional classifications using the WEGO software [75] to view the distribution of gene functions in *Litchi chinensis* at the macro level. For pathway-enrichment analysis, all unigenes were mapped to terms in the KEGG database. The datasets are available from the NCBI Short Read Archive (SRA) with the accession number SRX0255051. The assembled sequences have been stored in the NCBI’s Transcriptome Shotgun Assembly (TSA) database which can be searched for using the Gene-IDs listed in Additional file 9.

### DTA library preparation and sequencing

The total RNA of fruitlet samples collected at 1, 3, 5 and 7 days after treatment from both shaded and non-shaded trees, was isolated and pooled with equivalent quantities from the same treatment. Each DTA library included 12 pooled samples from four harvesting times (1, 3, 5 and 7 days after treatment) of three biological replicate trees. The sequence tag library preparation for two samples (non-shaded and shaded) was performed in parallel using the Gene Expression Sample Prep Kit (Illumina).

First, total RNA was used for mRNA purification with magnetic oligo(dT) beads. After reverse transcription and second-strand synthesis, the bead-bound double-stranded cDNA was subsequently digested with endonuclease *Nla*III which recognizes and cuts at the CATG sites on cDNA. These cDNA fragments with 3′ ends were then purified by magnetic beads precipitation and Illumina adaptor 1 was added to the 5′ ends. The junction of Illumina adaptor 1 and the CATG site is the recognition site of *Mme*I, which cuts 17 bp downstream of the CATG site, producing tags with adapter 1. After removing 3′ fragments with magnetic beads precipitation, Illumina adaptor 2 was introduced at 3′ ends of tags, forming a tag library. After 15 cycles of linear PCR amplification, 85 base strips were purified by PAGE gel electrophoresis. Finally, these strips were digested, and the single-chain molecules were fixed onto the Solexa Sequencing Chip (flow cell) for sequencing. The datasets are deposited in the NCBI’s SRA database with the accession numbers SRX258094 and SRX258095 for the shaded and non-shaded libraries, respectively.

### Analysis and mapping of DTA tags

Sequencing-received raw image data were transformed by base calling into raw reads. Clean tags were obtained by removing the adaptor sequence, empty tags (reads with only adaptor sequences but no tags), low-quality sequences (tags with unknown sequences ‘N’) and low-complexity sequences as well as tags with a copy number of 1 (probably due to sequencing error). All clean tags were mapped back to the litchi fruit transcriptome reference database by using SOAPaligner (Version 2.20) with a maximum of one nucleotide mismatch allowed at the parameters of “-m 0 -x 1000 -s 40 -l 35 -v 3 -r 2”. The meaning and selection principles of the parameters are available on the internet (http://soap.genomics.org.cn/soapaligner.html). Clean tags mapped to reference sequences, from multiple genes, were filtered and the remaining tags became unambiguous clean tags. For gene-expression analysis, the number of unambiguous clean tags was calculated and then normalized to TPM (Transcript Per Million clean tags) [76,77].

### Identification of differentially expressed unigenes

A rigorous algorithm was developed to identify differentially expressed unigenes between the two samples (non-shaded and shaded) based on a previously described method [32]. The FDR was controlled to determine differentially expressed genes [78]. In this study, FDR ≤0.001 and the absolute value of log_2_ ratio ≥1 (fold-change ≥2) was used as the threshold to judge the significance of unigene expression differences. For functional and pathway-enrichment analysis, all differentially expressed unigenes were mapped to terms in GO and the KEGG database. Unigenes with similar expression patterns usually mean functional correlation. We performed cluster analysis of unigene expression patterns with ‘cluster’ [79] software and ‘Java TreeView’ [80] software. The annotation of all significant unigenes was further supplemented with manual BLASTX, conserved domains, and literature searches. Using this combined information, a functionally driven classification was manually created.

### Quantitative real-time PCR analysis

Fourteen genes were randomly selected, from significant differentially expressed DTA tags between the two libraries, to observe their expression patterns during shading treatment by qRT-PCR. The list of gene-specific primers, designed on the 3′ untranslated gene region, is shown in Additional file 8. Purified total RNA (2 μg) from each sample was used to synthesize cDNA in a 20 μL reaction using the M-MLV first-strand synthesis system (Promega). Each qRT-PCR reaction was performed using 40 ng of cDNA in a 20 μL reaction using a SYBR Green-based PCR assay (TaKaRa). The quantitative reactions were performed on the ABI 7500 Real-Time PCR System with 7500 System Software (Applied Biosystems, USA). The comparative *C*t (threshold cycle) method of quantification was used with litchi *EF-1α* as the reference which was amplified in parallel with the target unigene(s), allowing unigene expression normalization and providing quantification according to our previous study [81]. The relative fold-change for each of the selected genes was determined for both the control and shaded trees. Three independent biological replicates of each sample and three technical replicates of each biological replicate were used for real-time PCR analysis. PCR amplifications included the following condition: 95°C for 1 min, followed by 40 cycles of 95°C for 5 s, 55°C for 30 s and 72°C for 30 s. Dissociation curves were run to determine the specificity of the amplification reactions. After validation tests, normalization to *EF-1α* was performed using the ΔΔ*C*t method (Applied Biosystems).

## Abbreviations

ABA: Abscisic acid; BLAST: Basic local alignment search tool; CFAR: Cumulative fruit abscission rate; COG: Clusters of orthologous groups; DTA: Digital transcript abundance; FDR: False discovery rate; GO: Gene ontology; KEGG: Kyoto encyclopedia of genes and genomes; NCBI: National center for biotechnology information; Q20 percentage: Percentage of bases whose quality was larger than 20 in clean reads; EST: Expressed sequence tags; FDR: False discovery rate; NR: Non-redundant; qRT-PCR: Quantitative real-time reverse transcriotion polymerase chain reaction; RNA-Seq: RNA sequencing; ROS: Reactive oxygen species.

## Competing interests

The authors declare that they have no competing interests.

## Authors’ contributions

This study was conceived by JGL. CQL and YW collected all organs and tissues of *Litchi chinensis*. The plant material preparation and gene-expression analyses were carried out by CQL and YW. CQL and JGL contributed to data analysis, bioinformatics analysis and manuscript preparation. JL was responsible for bioinformatics. XMH and HCW revised the manuscript. All authors read and approved the final manuscript.

## Supplementary Material

Additional file 1 **Length distributions of assembled contigs, scaffolds and unigenes.** The number of transcriptome assemblies in each size category is shown. Click here for file

Additional file 2 **Gap distribution of assembled scaffolds and unigenes.** Gap distribution (N/size) %: gap percentage (N amount/sequence length) distribution. Click here for file

Additional file 3 **Overview of the digital transcript abundance (DTA) profile.** DTA results for the shaded and non-shaded libraries. Click here for file

Additional file 4 **Distribution of total tags and clean tags over different tag abundance categories.** (A) Distribution of total clean tags. Numbers in square brackets show the range of copy numbers for a specific category of tags. Numbers in parentheses indicate the total tag copy number and ratio for all the tags in that category. (B) Distribution of distinct clean tags. Numbers in square brackets show the range of copy numbers for a specific category of tags. Numbers in parentheses indicate the total types of tags in that category. Click here for file

Additional file 5 **Vector graph of the distribution of the ratio of tag expression between shaded and non-shaded libraries.** The x-axis represents the fold-change of differentially expressed unique tags between the shaded and non-shaded libraries. The y-axis represents the number of unique tags (log10). Differentially accumulating unique tags with a fivefold difference between libraries are shown in the red region (99.35%). The green (0.43%) and blue (0.20%) regions represent unique tags that are up- or down-regulated, respectively, by more than five-fold in the shaded library. Click here for file

Additional file 6 **Differential expression analysis and clustering analysis of digital transcript abundances (DTAs).** (**A**) Differential expression analysis of unigenes. We used a false discovery rate (FDR) <0.001 and the absolute value of log_2_ ratio ≥1 as the threshold to judge the significance of transcript abundance differences. Red dots represent transcripts that were more prevalent in the shaded library. Green dots show those that were present at a lower frequency after shading treatment, while blue dots indicate transcripts that did not change significantly. (**B**) Clustering analysis of differential gene-expression patterns. TPM: transcript copies per million tags. Click here for file

Additional file 7 **Functional categorization of significantly and differentially expressed unigenes for shading-treated fruitlet.** In this table, a total of 1,039 significantly and differentially expressed unigenes are divided into 14 groups (photosynthesis, carbohydrate metabolism, transport, transcription factors, hormone response, stress response, cell wall modification, hydrolysis and cell death, reactive oxygen species, cell cycle, development, DNA/RNA and unknown) based on the functional annotation (NR database). Both FDR (false discovery rate) <0.001 and an absolute value of log_2_ ratio ≥1 was used as the threshold to judge the significance of gene-expression differences. Click here for file

Additional file 8 **Gene-specific primers of 14 randomly selected genes used in qRT-PCR analysis.** This table lists all of the primers used in qRT-PCR analysis. Sequence length, annotation of the top BLAST hits in the NCBI non-redundant (NR) database and the homologous genes (with corresponding *E*-values ) are shown. FDR: false discovery rate. Click here for file

Additional file 9 Assembled sequences in the litchi transcriptome that are annotated in the NCBI non-redundant (NR) database. Click here for file
