# Supplementary figures and images for: De novo assembly and characterization of fruit transcriptome in Litchi chinensis Sonn and analysis of differentially regulated genes in fruit in response to shading
Source: BMC Genomics. 2013 Aug 14;14:552. doi: 10.1186/1471-2164-14-552 (PMC3751308; doi:10.1186/1471-2164-14-552)

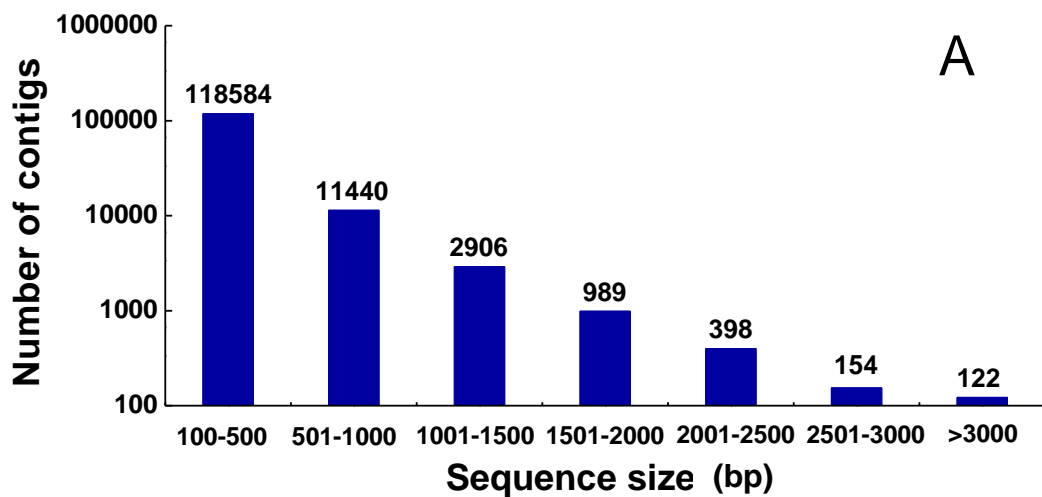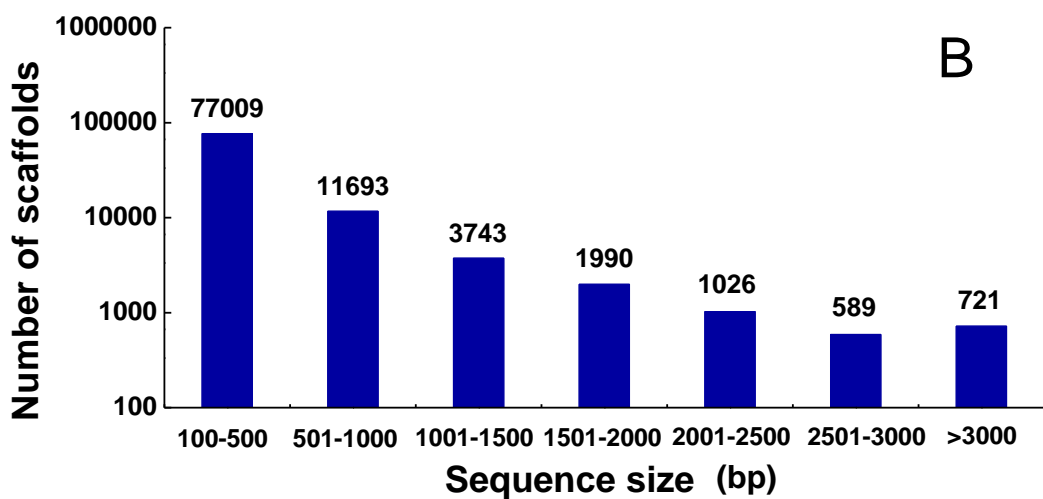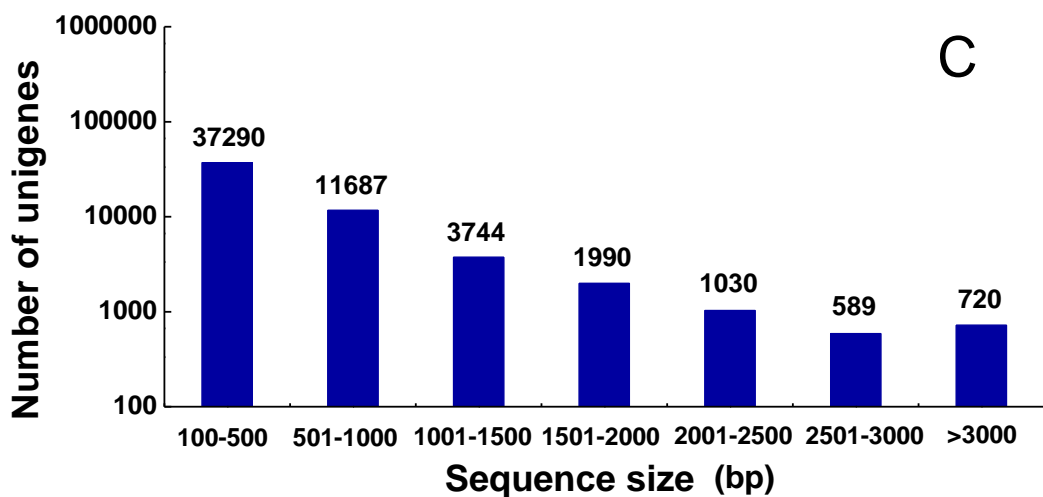

Supplement: Additional file 1 — Length distributions of assembled contigs, scaffolds and unigenes. The number of transcriptome assemblies in each size category is shown. [file 1471-2164-14-552-S1.pdf]

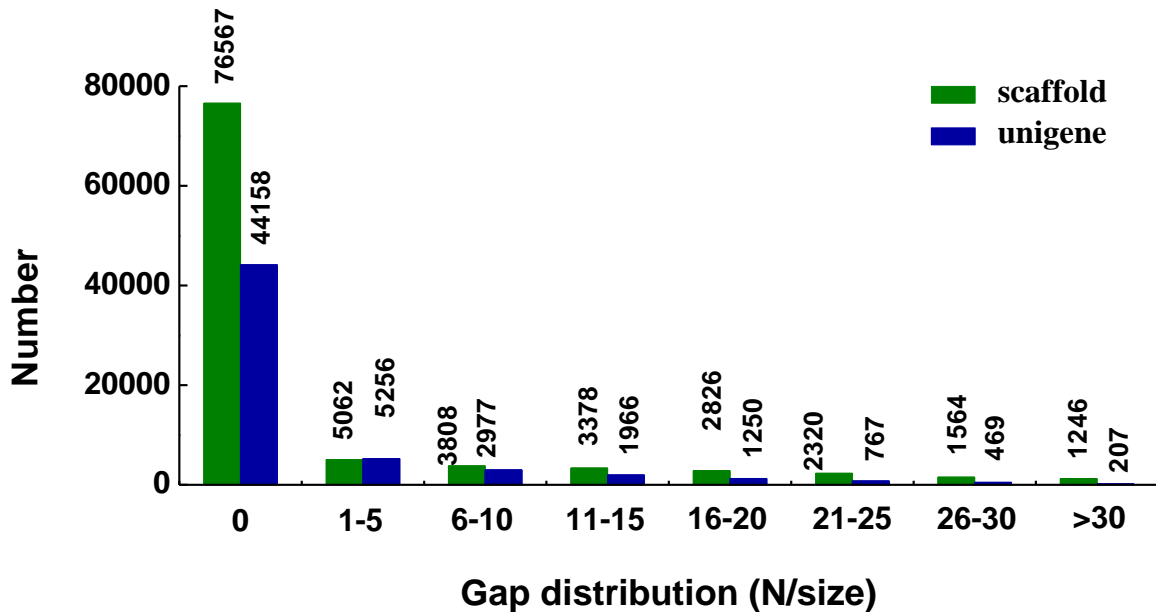

Supplement: Additional file 2 — Gap distribution of assembled scaffolds and unigenes. Gap distribution (N/size) %: gap percentage (N amount/sequence length) distribution. [file 1471-2164-14-552-S2.pdf]

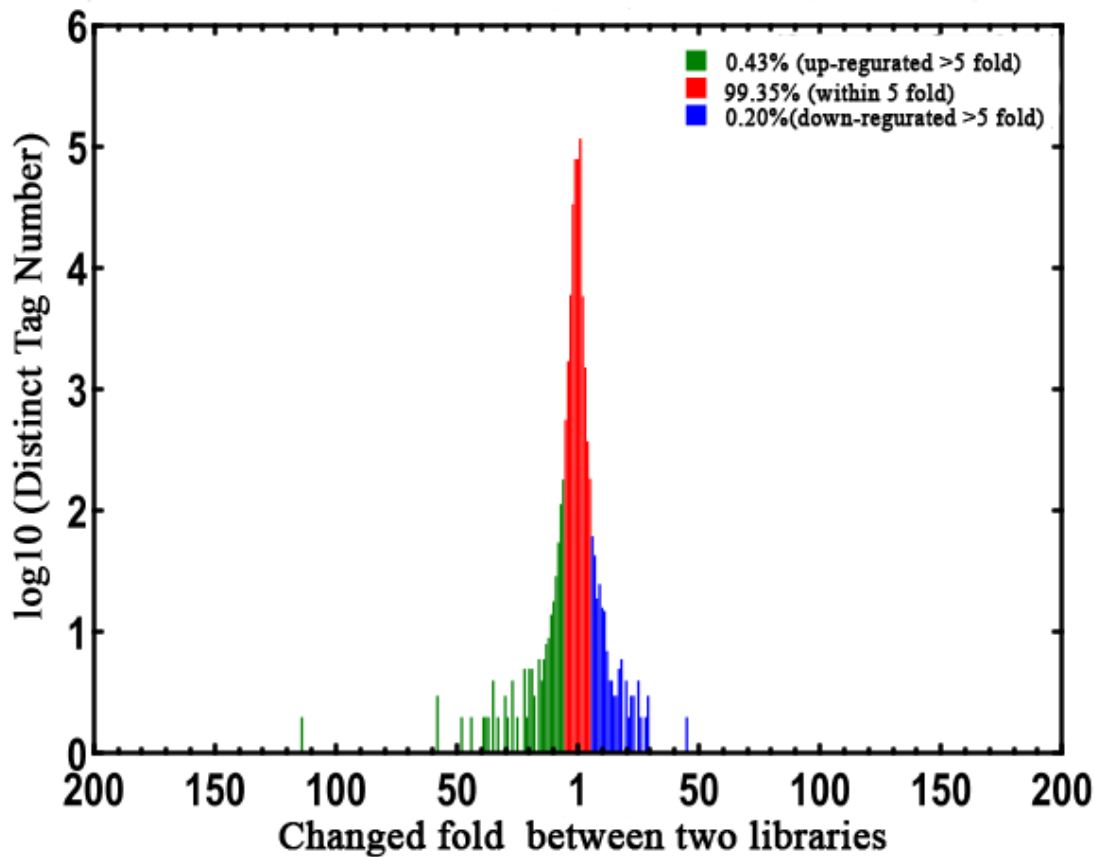

Supplement: Additional file 5 — Vector graph of the distribution of the ratio of tag expression between shaded and non-shaded libraries. The x-axis represents the fold-change of differentially expressed unique tags between the shaded and non-shaded libraries. The y-axis represents the number of unique tags (log10). Differentially accumulating unique tags with a fivefold difference between libraries are shown in the red region (99.35%). The green (0.43%) and blue (0.20%) regions represent unique tags that are up- or down-regulated, respectively, by more than five-fold in the shaded library. [file 1471-2164-14-552-S5.pdf]

**A**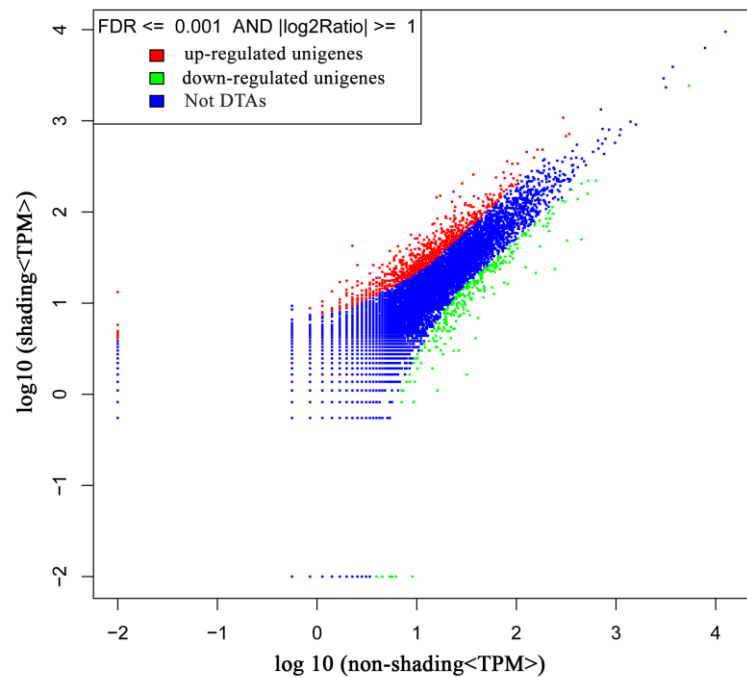**B**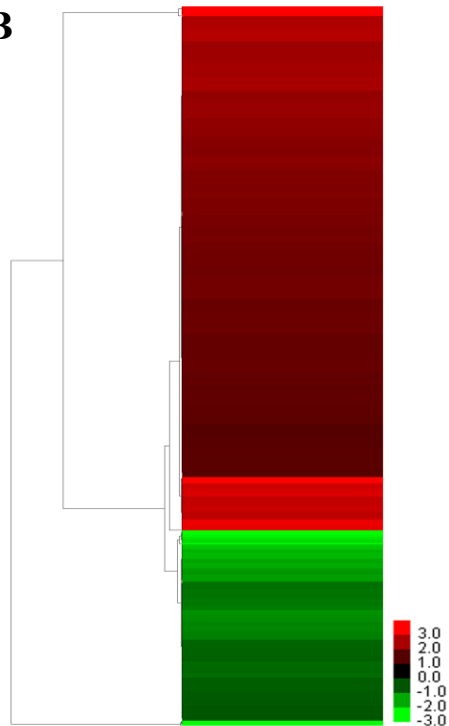

Supplement: Additional file 6 — Differential expression analysis and clustering analysis of digital transcript abundances (DTAs). (A) Differential expression analysis of unigenes. We used a false discovery rate (FDR) <0.001 and the absolute value of log2 ratio ≥1 as the threshold to judge the significance of transcript abundance differences. Red dots represent transcripts that were more prevalent in the shaded library. Green dots show those that were present at a lower frequency after shading treatment, while blue dots indicate transcripts that did not change significantly. (B) Clustering analysis of differential gene-expression patterns. TPM: transcript copies per million tags. [file 1471-2164-14-552-S6.pdf]
