# Supplementary material for: De novo assembly and characterization of fruit transcriptome in Litchi chinensis Sonn and analysis of differentially regulated genes in fruit in response to shading
Source: BMC Genomics. 2013 Aug 14;14:552. doi: 10.1186/1471-2164-14-552 (PMC3751308; doi:10.1186/1471-2164-14-552)
Supplement: Additional file 4 — Distribution of total tags and clean tags over different tag abundance categories. (A) Distribution of total clean tags. Numbers in square brackets show the range of copy numbers for a specific category of tags. Numbers in parentheses indicate the total tag copy number and ratio for all the tags in that category. (B) Distribution of distinct clean tags. Numbers in square brackets show the range of copy numbers for a specific category of tags. Numbers in parentheses indicate the total types of tags in that category. [file 1471-2164-14-552-S4.pdf]

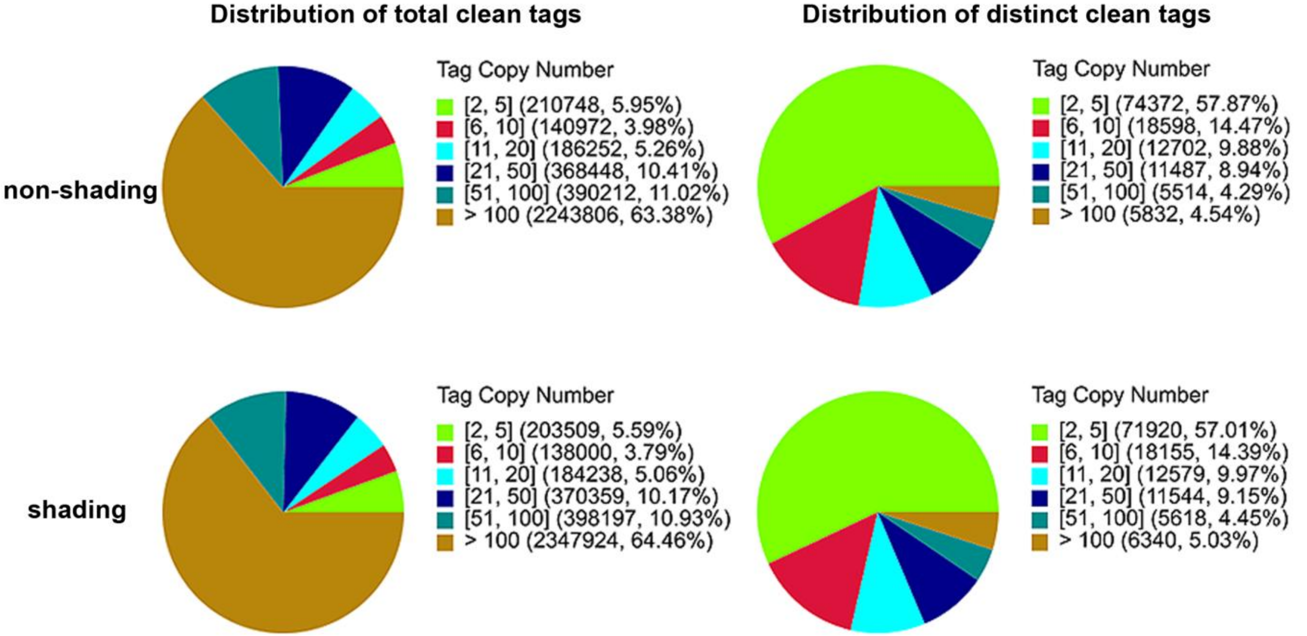

Distribution of total clean tags
Distribution of distinct clean tags
non-shading
Tag Copy Number
[2, 5] (210748, 5.95%)
[6, 10] (140972, 3.98%)
[11, 20] (186252, 5.26%)
[21, 50] (368448, 10.41%)
[51, 100] (390212, 11.02%)
> 100 (2243806, 63.38%)
Tag Copy Number
[2, 5] (74372, 57.87%)
[6, 10] (18598, 14.47%)
[11, 20] (12702, 9.88%)
[21, 50] (11487, 8.94%)
[51, 100] (5514, 4.29%)
> 100 (5832, 4.54%)
shading
Tag Copy Number
[2, 5] (203509, 5.59%)
[6, 10] (138000, 3.79%)
[11, 20] (184238, 5.06%)
[21, 50] (370359, 10.17%)
[51, 100] (398197, 10.93%)
> 100 (2347924, 64.46%)
Tag Copy Number
[2, 5] (71920, 57.01%)
[6, 10] (18155, 14.39%)
[11, 20] (12579, 9.97%)
[21, 50] (11544, 9.15%)
[51, 100] (5618, 4.45%)
> 100 (6340, 5.03%)
